# ST218 *Klebsiella pneumoniae* became a high-risk clone for multidrug resistance and hypervirulence

**DOI:** 10.1186/s12866-024-03205-8

**Published:** 2024-02-12

**Authors:** Ping Yang, Chao Liu, Pengcheng Du, Juan Yi, Zhenchao Wu, Jiajia Zheng, Ning Shen, Liyan Cui, Ming Lu

**Affiliations:** 1 Department of Pulmonary and Critical Care Medicine, Peking University Third Hospital, Beijing, China; 2 Institute of Medical Technology, Peking University Health Science Center, Beijing, China; 3 Department of Infectious Diseases, Peking University Third Hospital, Beijing, China; 4 Center of Infectious Disease, Peking University Third Hospital, Beijing, China; 5 Qitan Technology Ltd., Chengdu, China; 6 Department of Laboratory Medicine, Peking University Third Hospital, Beijing, China

**Keywords:** Hypervirulent *Klebsiella pneumoniae*, Multidrug resistant, ST218, *bla*_NDM-1_

## Abstract

**Background:**

The occurrence of multidrug-resistant and hypervirulent *Klebsiella pneumoniae* (MDR-hvKp) worldwide poses a great challenge for public health. Few studies have focused on ST218 MDR-hvKp.

**Methods:**

Retrospective genomic surveillance was conducted at the Peking University Third Hospital from 2017 and clinical information was obtained. To understand genomic and microbiological characteristics, antimicrobial susceptibility testing, plasmid conjugation and stability, biofilm formation, serum killing, growth curves and whole-genome sequencing were performed. We also assessed the clinical and microbiological characteristics of ST218 compared with ST23.

**Results:**

A total of eleven ST218 Kp isolates were included. The most common infection type was lower respiratory tract infection (72.7%, 8/11) in our hospital, whereas ST23 hvKp (72.7%, 8/11) was closely associated with bloodstream infection. Notably, nosocomial infections caused by ST218 (54.5%, 6/11) was slightly higher than ST23 (36.4%, 4/11). All of the ST218 and ST23 strains presented with the virulence genes combination of *iucA + iroB + peg344 + rmpA + rmpA2*. Interestingly, the virulence score of ST218 was lower than ST23, whereas one ST218 strain (pPEKP3107) exhibited resistance to carbapenems, cephalosporins, β-lactamase/inhibitors and quinolones and harbored an ~ 59-kb IncN type MDR plasmid carrying resistance genes including *bla*_NDM-1_, *dfrA14* and *qnrS1*. Importantly, *bla*_NDM-1_ and *qnrS1* were flanked with IS*26* located within the plasmid that could successfully transfer into *E. coli J53*. Additionally, PEKP2044 harbored an ~ 41-kb resistance plasmid located within *tetA* indicating resistance to doxycycline.

**Conclusion:**

The emergence of *bla*_NDM-1_ revealed that there is great potential for ST218 Kp to become a high-risk clone for MDR-hvKp, indicating the urgent need for enhanced genomic surveillance.

**Supplementary Information:**

The online version contains supplementary material available at 10.1186/s12866-024-03205-8.

## Introduction

Hypervirulent *Klebsiella pneumoniae* (hvKp), a pathotype of *Klebsiella pneumoniae*, was first reported in Taiwan and is a common pathogen for community-acquired pyogenic liver abscesses, distinguishing it from classical *Klebsiella pneumoniae* (cKp), which is usually multidrug resistant (MDR) and is highly associated with nosocomial infection [1–3].

Previously, hvKp was defined as hypermucoviscosity and confirmed by string test positivity [4]. Thomas and his colleagues found that aerobactin plays important roles in hypervirulence [5]. Recently, the combination of *rmpA, rmpA2, iucA, iroB* and *peg-344* was confirmed, representing higher accuracy for defining hvKp [6]. Importantly, epidemiological investigations have demonstrated that hvKp is rapidly increasing and tends to replace cKp as the dominant pathogen in hospital- and healthcare-associated infections [7].

Even more worrisome is the continuing occurrence of multidrug resistant and hypervirulent Kp (MDR-hvKp) worldwide, which is virtually impossible to treat, increasing the burden on public health [1, 8–10]. Previous studies reported that the acquisition of virulence-associated genes within cKp and acquisition of MDR plasmids by HvKp primarily contributed to MDR-hvKp, especially in China [7, 11]. ST23 is a classical representation of hvKp. Our previous study also reported that mobile genetic elements like plasmids and IS*26-*flanked resistance islands play crucial roles in shaping ST23 MDR-hvKp [12]. ST218 is a single-locus variant of ST23 that initially harbored virulence-associated genes and caused severe systemic infections but has never been systematically reported before [13].

Here, we reported for the first time that hypervirulent ST218 Kp acquired transferable *bla*_NDM-1_, which conferred MDR and hypervirulence phenotypes. Compared with ST23, we found that the genomic characteristics of ST218 are similar to those of ST23 regarding virulence and plasmid, whereas ST218 tends to acquire carbapenemases.

## Methods

### Enrolled ST218 Kp strains

We performed a genomic surveillance at the Peking University Third Hospital from 2017 to 2022, 1381 *Klebsiella. spp* were obtained, among which eleven ST218 Kp isolates (0.80%, 11/1381) were detected and further analyzed. The Vitek automated compact 2 system was used to identify the Kp isolates.

The clinical data of the infected patients was collected. Basic demographic information, specimens, infection type, antimicrobial agent exposure within 90 days, invasive incubation, metastatic infection and outcome in 30 days were collected. Additionally, the Charlson comorbidity index (CCI) and sequential organ failure assessment (SOFA) score were calculated. Community-acquired infections (CAIs) and hospital-acquired infections (HAIs) were identified as previously described [7]. Metastatic infection was defined as the presence of > 1 infection site in the same patient [14].

### Antimicrobial susceptibility testing (AST)

AST was performed using the Vitek 2 system. The antimicrobial agents contained cefepime, ceftazidime, aztreonam, imipenem, meropenem, piperacillin/tazobactam, cefoperazone-sulbactam, amikacin, tobramycin, levofloxacin, ciprofloxacin, minocycline, tigecycline and trimethoprim/sulfamethoxazole. The results were interpreted according to 2021 Clinical and Laboratory Standards Institute (CLSI) guideline breakpoints, and the breakpoints for tigecycline based on the EUCAST recommendation as previously described [15]. MDR was defined as the resistance to three or more different antimicrobial categories.

### String test and mucosviscosity

The hypermucoviscous phenotype was evaluated by the string test as described previously [16]. Briefly, the Kp strains were inoculated onto Columbia agar with sheep blood (PB0123A, OXOID, Beijing, China) and cultured at 37 °C overnight. A string test was considered positive when a viscous string > 5 mm in length was generated by touching and pulling a single colony upward using a bacteriology inoculation loop.

Mucosviscosity was evaluated as previously described [17]. Briefly, Kp strains were inoculated in LB with an overnight incubation. The absorbance was measured at OD600 (preOD600). Then, 1000 μL of culture was centrifuged at 2000×g for 5 min, and the OD600 of the supernatant was evaluated (post OD600). The post/pre OD600 ratio represented the mucoviscosity.

### Plasmid stability and conjugation assay

We performed a conjugation assay to evaluate the transferability of the plasmid carrying *bla*_NDM-1_. The recipient strain was classical *E. coli J53*, and we mixed the PEKP3107 (carrying *bla*_NDM-1_) and *E. coli J5* in a 1:1 ratio and cultured in LB broth overnight [18]. Then, the MacConkey agar with a concentration of 2 mg/liter meropenem and 200 mg/liter sodium azide was prepared, on which the above mixture was further inoculated. We finally identified the transconjugants after overnight incubation. The transconjugants were evaluated by AST. Furthermore, we continuously passaged the isolates carrying *bla*_NDM-1_ and *tet(A)* to the 10th generation and performed AST to clarify the stability of the plasmid.

### Growth curve

We selected monoclones and cultured them overnight at 37 °C. The suspension of 0.5 McF was prepared with 0.45% NaCl and then added to a 96-well plate with 3 triplicates of 10 μL per well. We further added 190 μL of LB broth per well and cultured these isolates. Finally, the absorbance at OD590 was continuously recorded by the Tecan infinite 200Pro every hour until 12 h.

### Serum killing assay

The 0.5 McF bacterial solution was prepared and diluted to a final concentration of 1 × 10^6^ CFU/ml, 25 μL of which was added to 75 μL of serum collected from healthy adults. The mixture (1 μL) was spotted on the blood plate at 0 min, 60 min, 120 min, and 180 min and cultured overnight. Viable counts were recorded. If the number of live bacteria was more than 100% after 3 hours, it was resistant, and if the opposite occurred, it was sensitive.

### Biofilm formation assay

Ten microliters of 0.5 McF bacterial solution and 190 μL of LB broth were added to a 96-well plate thrice and cultured for 24 hours at 37 °C. The supernatant was absorbed and discarded, washed repeatedly with distilled water. Subsequently, 200 μL of 0.1% crystal violet dye was added, and further washed three times after 20 minutes. Finally, 200 μL of 95% ethanol was added, and the absorbance value was measured and recorded at OD 590 nm. The results were interpreted based on the absorbance value compared to the average value of the well with only LB broth (Ac) as previously described [11].

### *Galleria mellonella* larvae lethality assay

The *G. mellonella* larvae lethality assay was performed as described previously [19]. Briefly, the bacterial solution of 1 × 10^8^ CFU/ml was prepared, 10 μl of which was injected into ten larvae in each group. The infected larvae were incubated at 37 °C, and mortality was observed every 12 h for 3 days.

### Whole genome sequencing (WGS) and bioinformatic analysis

First, Kp DNA was extracted for next-generation sequencing, and the preparation of libraries using Nextera technology [7]. Paired-end reads of 150 bp were generated by the Illumina NovaSeq 6000 platform was used for sequencing (paired end reads, 150 bp), and isolates PEKP3107 and PEKP2044 were selected for nanopore sequencing. To assembly the complete genomes, a hybrid de novo assembly strategy using Unicycler v0.4.4 was employed to combine highly accurate short reads and long reads, which were obtained from Illumina sequencing and Nanopore sequencing, respectively [20]. Additionally, we collected the previously published genome data of ST218 *K. pneumoniae* from the NCBI database (up to September 1, 2021). Prokka software was used for primary gene prediction and initial annotation [21]. In addition, Kleborate software was utilized to identify sequence types and capsule serotypes [22]. Resistance genes, virulence genes, IS sequences and plasmid replicon types were further detected by BLAST through comparison with the ResFinder [23], Virulence Factor Database [24], IsFinder [25], and plasmidFinder [26] databases.

For phylogenetic analysis, we mapped the Illumina sequencing reads to the reference (ST23 *K. pneumoniae* NTUH_K2044) using Bowtie 2 v2.2.8 [27]. The single nucleotide polymorphisms (SNPs) were analyzed by Samtools v1.9. We used the iSNV calling pipeline (https://github.com/generality/iSNVcalling) to combine the SNP sites of all the ST218 Kp strains according to the reference genome (*K. pneumoniae* NTUH_K2044). The high-quality SNPs were collected, and the recombination sites were removed using Gubbins v2.4.1 [28]. Finally, we identified the concatenated sequences of filtered polymorphic sites that were conserved in all genomes, defined as core genome SNPs (cgSNPs), and the phylogenetic analysis was then conducted using FastTree software by the maximum likelihood method [29].

## Results

### Emergence of MDR hypervirulent ST218 *Klebsiella pneumoniae*

During the six-year surveillance, we obtained eleven ST218 Kp isolates from eleven patients out of 1381 Klebsiella isolates in total. Notably, one of the eleven Kp strains (9.1%, 1/11), PEKP3107, represented an MDR phenotype was resistant to carbapenems, cephalosporins, β-lactamase/inhibitors and quinolones (Table 1). This strain was isolated from the urine sample of a 52-year-old male patient who experienced a community-acquired infection and exposed antibiotics within 90 days. The urinary catheter and gastrostomy tube were indwelled within the patient who presented with various underlying diseases (CCI = 3) (Table 2).

**Table 1:** Antibiotic sensitivity patterns and hypermucoviscosity phenotype of ST218 *Klebsiella pneumoniae* strains and its transconjugant

Strain	PEKP2044	PEKP3093	PEKP1026	219	PE262	PE388	PE393	PE5196	PE5210	297	PEKP3107	Transconjugant	PEKP3107 10th	PEKP2044 10th
String test	positive	positive	positive	positive	positive	positive	positive	positive	positive	positive	positive		positive	positive
piperacillin/tazobactam	≤4	≤4	≤4	≤4	≤4	8	≤4	≤4	≤4	≤4	≥128	≥128	≥128	≤4
ceftazidime	≤0.12	≤0.12	≤0.12	≤0.12	≤0.12	0.5	≤0.12	≤0.12	≤0.12	≤0.12	≥64	≥64	≥64	≤0.12
cefoperazone/sulbactam	≤8	≤8	≤8	≤8	≤8	≤8	≤8	≤8	≤8	≤8	≥64	≥64	≥64	≤8
cefepime	≤0.12	≤0.12	≤0.12	≤0.12	≤0.12	≤0.12	≤0.12	≤0.12	≤0.12	≤0.12	≥32	16	≥32	≤0.12
aztreonam	≤1	≤1	≤1	≤1	≤1	≤1	≤1	≤1	≤1	≤1	≤1	≤1	≤1	≤1
imipenem	0.5	≤0.25	≤0.25	≤0.25	0.5	0.5	≤0.25	≤0.25	≤0.25	0.5	≥16	≥16	≥16	0.5
meropenem	≤0.25	≤0.25	≤0.25	≤0.25	≤0.25	≤0.25	≤0.25	≤0.25	≤0.25	≤0.25	≥16	≥16	≥16	≤0.25
amikacin	≤2	≤2	≤2	≤2	≤2	≤2	≤2	≤2	≤2	≤2	≤2	≤2	≤2	≤2
tobramycin	≤1	≤1	≤1	≤1	≤1	≤1	≤1	≤1	≤1	≤1	≤1	≤1	≤1	≤1
ciprofloxacin	≤0.25	≤0.25	≤0.25	≤0.25	≤0.25	≤0.25	≤0.25	≤0.25	≤0.25	≤0.25	1	1	1	≤0.25
levofloxacin	≤0.12	≤0.12	≤0.12	≤0.12	1	≤0.12	≤0.12	≤0.12	≤0.12	≤0.12	1	1	1	≤0.12
doxycycline	≥16	1	1	1	1	2	1	1	1	2	1	1	1	≥16
minocycline	4	2	2	2	2	4	2	2	≤1	4	2	≤1	2	4
tigecycline	≤0.5	1	1	1	1	1	≤0.5	1	≤0.5	1	1	≤0.5	1	1
trimethoprim/sulfamethoxazole	≤20	≤20	≤20	≤20	≤20	≤20	≤20	≤20	≤20	≤20	40	≥320	40	≤20

**Table 2:** Clinical characteristics of ST218 vs. ST23 *Klebsiella pneumoniae*

ST	ID	Department	Sex	Age	Specimen	Infection Type	^**a**^CCI	Antibiotic exposure within 90 days	Central intravenous catheter	Urinary catheter	Endotracheal tube	Gastrostomy tube	Drainage tube	Metastatic infection	^**b**^SOFA	Outcome in 30 Days
ST218	PEKP3107	Emergency	Male	52	Urine	^c^CAI	3	^e^Y	^f^N	Y	N	Y	N	N	0	Survive
ST218	PEKP2044	Thoracic surgery	Male	35	Sputum	^d^HAI	0	N	N	Y	N	N	N	N	0	Survive
ST218	PEKP1026	Hematology	Male	30	Abdominal fluid	HAI	4	Y	N	N	N	N	N	N	0	Survive
ST218	219	Nephrology	Female	56	Urine	CAI	2	N	N	N	N	N	N	N	0	Survive
ST218	PEKP3093	Cardial Surgery	Male	68	Sputum	HAI	1	N	Y	Y	N	N	Y	N	0	Survive
ST218	297	oncology radiotherapy	Male	66	Sputum	CAI	6	N	N	N	N	N	N	N	0	Survive
ST218	PE262	general surgery	Male	52	Sputum	CAI	3	N	N	N	N	N	N	N	2	Survive
ST218	PE388	Respiratory medicine	Male	64	Sputum	CAI	2	N	N	N	N	N	N	N	0	Survive
ST218	PE393	Neurology	Female	82	Sputum	HAI	1	N	N	Y	N	Y	N	Y	1	Survive
ST218	PE5196	neurosurgery	Male	57	Sputum	HAI	4	N	N	N	N	N	N	N	0	Survive
ST218	PE5210	Cardial Surgery	Male	46	Sputum	HAI	3	N	N	N	N	N	N	N	0	Survive
ST23	PEKP1028	Emergency	Male	49	Blood	CAI	2	N	N	N	N	N	N	N	0	Survive
ST23	PEKP1047	immunology	Female	66	Sputum	HAI	3	N	N	N	N	N	N	N	0	Survive
ST23	PEKP1050	Emergency	Male	91	Blood	HAI	2	Y	N	N	N	N	N	N	2	Survive
ST23	PEKP1064	infectious disease	Female	63	Puncture fluid	HAI	1	Y	N	N	N	N	Y	N	0	Survive
ST23	PEKP1083	Emergency	Male	70	Blood	CAI	3	Y	N	N	N	N	Y	Y	6	Survive
ST23	PEKP1095	Respiratory medicine	Female	66	Sputum	HAI	0	N	N	N	N	N	N	N	0	Survive
ST23	PEKP1119	Emergency	Female	76	Blood	CAI	5	Y	N	N	N	N	N	N	3	Survive
ST23	PEKP1132	Emergency	Male	38	Blood	CAI	2	Y	N	N	N	N	N	N	2	Survive
ST23	PEKP2063	Emergency	Male	82	Blood	CAI	0	N	N	N	N	N	N	N	1	Survive
ST23	PEKP2002	Emergency	Male	37	Blood	CAI	1	Y	N	N	N	N	N	N	2	Survive
ST23	PEKP2074	infectious disease	Female	65	Blood	CAI	3	N	N	N	N	N	N	N	1	Survive

^a^CCI, Charlson comorbidity index

^b^SOFA, Sequential organ failure assessment

^c^CAI, Community acquired infections

^d^HAI, Hospital acquired infections

^e^Y, Yes

^f^N, No

In addition, another isolate named PEKP2044 presented resistance to doxycycline. This strain was isolated from sputum from a 35-year-old male patient who experienced nosocomial infection with urinary catheter intubation. In addition, none of the other nine strains presented resistance to any of the tested antimicrobial agents. All eleven isolates presented a hypermucoviscous phenotype (Table 1).

### Features of resistance genes, plasmid types and virulence gene profiles

To further clarify the genomic traits of ST218 Kp, we collected a total of 29 ST218 Kp genomes, consisting of eighteen previously published and eleven we sequenced.

**Fig. 1:**
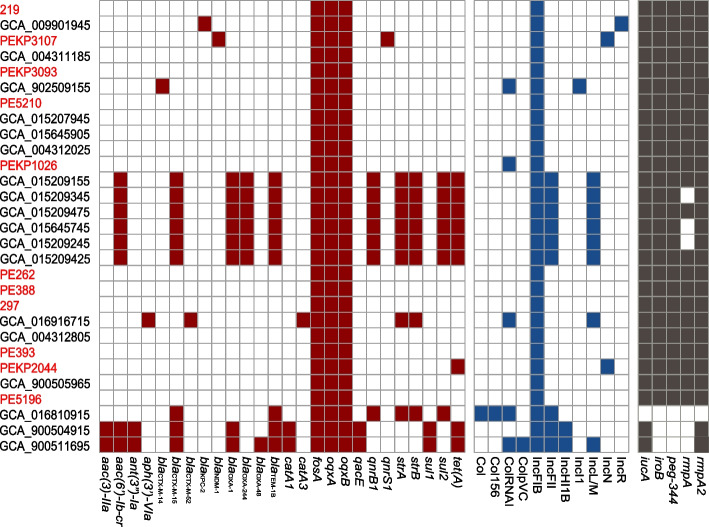
Global distribution of genomic characteristics of the ST218 *Klebsiella pneumoniae* isolates. Strains isolated from our hospital are in red text. Red: antimicrobial resistance genes; blue: plasmid types; dark gray: virulence genes

All ST218 isolates were found to possess *fosA* and *oqxAB*, which are associated with resistance to fosfomycin and quinolones. However, it should be noted that *fosA* and *oqxAB* may not necessarily confer resistance to these antibiotics in *K. pneumoniae* [30, 31]. Notably, the majority of the ST218 strains harbored few resistance genes. Among the 29 total ST218 Kp strains, eleven isolates (37.9%, 11/29) were predicted to be multidrug resistant. Most MDR strains harbored *aac(6′)-Ib-cr, bla*_CTX-M-15_*, bla*_OXA-1_*, bla*_OXA-244_*, bla*_TEM-1B_*, qnrB1, strA, strB, sul2* and *tet(A)*. Among our 11 isolates, PEKP3107 was predicted to be MDR and presented resistance genes including *bla*_NDM-1_ and *qnrS1* and exhibited penicillin, cephalosporin, carbapenem and quinolone resistance. PEKP2044 harbored the resistance gene *tet(A)* and was resistant to tetracyclines (Fig. 1).

All 29 strains harbored the IncFIB type plasmid. Nine strains (31.0%, 9/29) carried the IncFII type plasmid, and eight strains (27.6%, 8/29) harbored the IncL/M plasmid replicon. The key virulence gene, *iucA*, was detected in 28 isolates (96.6%). A significant number of strains (92.9%, 26/29) harbored the salmochelin-associated gene (*iroB*) and *peg-344*. *RmpA* and *rmpA2* were present in 23 and 28 isolates (79.3 and 96.6%), respectively. A majority of ST218 strains presented with the combination of *iucA + iroB + peg344 + rmpA + rmpA2* (79.3%, 23/29). Only one isolate did not harbor the above five virulence genes (Fig. 1). Furthermore, we calculated that the SNP differences among these ST218 Kp isolates varied between 152 and 313, indicating that these strains were sporadic (Additional file 1: Table S1).

### Coharbouring of virulence plasmid and resistance plasmid

**Fig. 2:**
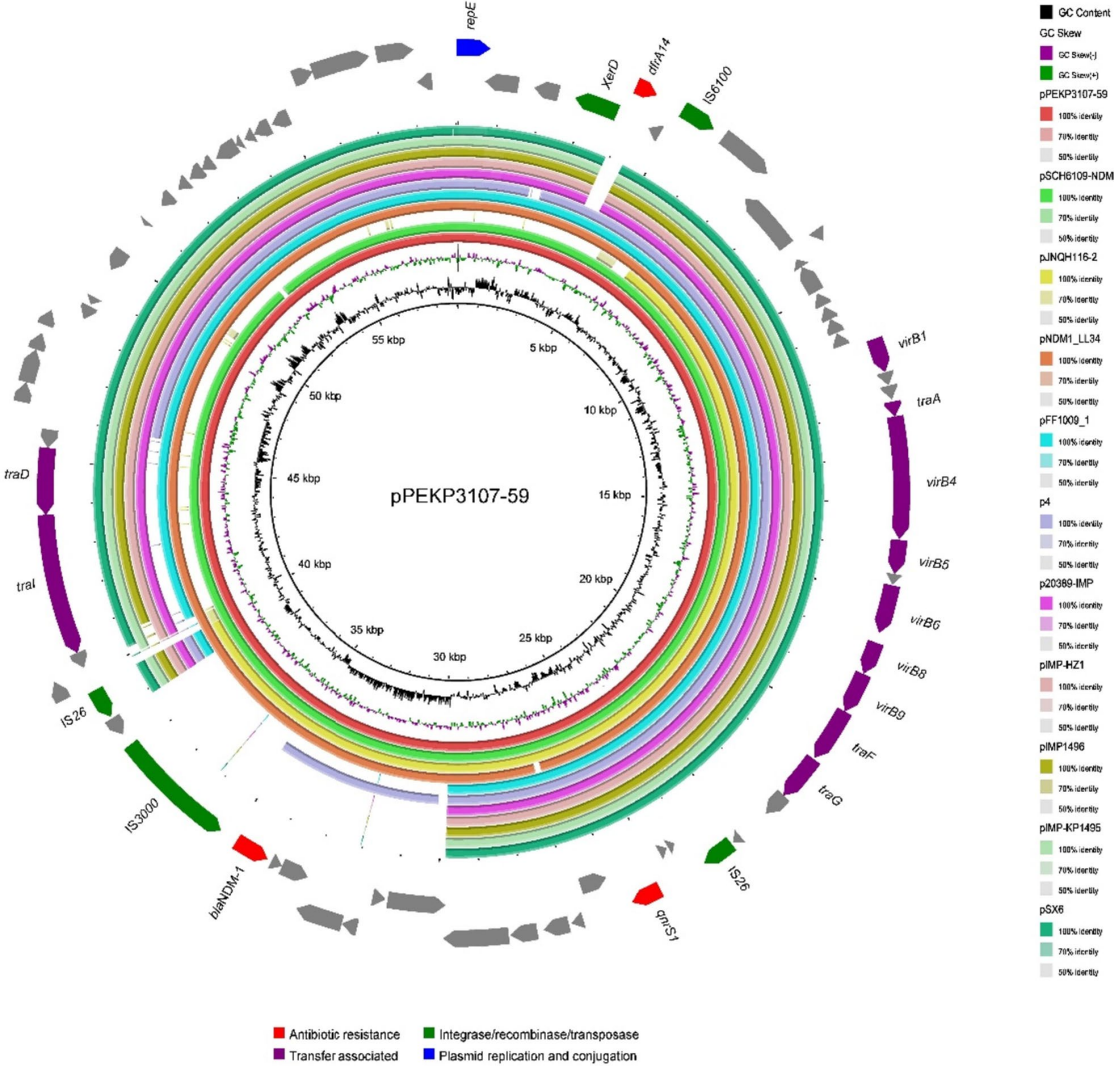
Comparison between plasmid pPEKP3107–59 found in this study and similar plasmids found in the online NCBI database. The outermost circle of arrows indicates the genes of the reference plasmid pPEKP3107–59 used for comparison (red: antimicrobial resistance genes; green: integrase, recombinase, and transposase genes; purple: transfer-associated genes; blue: plasmid replication and conjugation; gray: genes of other functions)

**Fig. 3:**
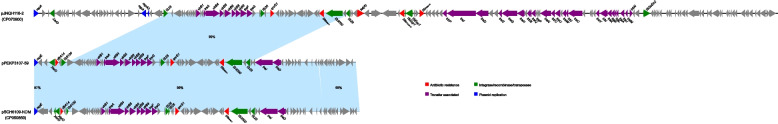
Alignments of plasmid pPEKP3107–59. The matched regions between two sequences are displayed by light blue blocks, and the identities are marked (red: antimicrobial resistance genes; green: integrase, recombinase, and transposase genes; purple: transfer-associated genes; blue: plasmid replication and conjugation; gray: genes of other functions)

In addition to the virulence plasmids, an ~ 59-kb MDR IncN type plasmid was also detected in the MDR isolate PEKP3107 we obtained. This plasmid, pPEKP3107–59, contained resistance genes including *bla*_NDM-1_, *dfrA14* and *qnrS1*. This plasmid conferred to multiple resistances, including resistances to β-lactams, carbapenems, and quinolones. Moreover, *bla*_NDM-1_ and *qnrS1* were located within the resistance element composed of IS*26*, IS*3000*, *bla*_NDM-1_, *qnrS1* and IS*26* (Figs. 2 and 3). pPEKP3107–59 showed high similarity to other *bla*_NDM-1_-carrying plasmids, such as pSCH6109-NDM (accession no. CP050859) and pJNQH116–2 (accession no. CP070900) (Fig. 3). Importantly, this IncN-type plasmid encoding *bla*_NDM-1_ could successfully transfer into *E. coli J53* by conjugation, indicating the transferability of the MDR plasmid.

Furthermore, an ~ 41-kb IncN type resistance plasmid was identified within the isolate PEKP2044. This plasmid, pPEKP2044–41 carried the resistance gene *tet(A)*, which conferred resistance to tetracyclines; *tet(A)* was located within the resistance element comprised of *traG*, *tet(A)*, *traI* and *traD* (Additional file 2: Figure S1). In addition, the two resistance plasmids (pPEKP3107–59 and pPEKP2044–41) remained stable after the 10th passage (Table 1).

### Clinical and genomic characteristics of ST23 vs. ST218

To further explore the characteristics of ST218, a single variant of ST23, we matched ST23 as a control. The age range of the ST218-infected patients was 30 y to 82 y, while those infected with ST23 Kp ranged from 37 y to 91 y. In the ST218 group, eight patients suffered from respiratory infections (72.7%, 8/11). A significant number of the ST23 Kp strains were isolated from the blood (72.7%, 8/11), followed by sputum (18.1%, 2/11) and puncture fluid (9.1%, 1/11). Interestingly, over half of the ST218 (54.5%, 6/11) cases were defined as HAIs, whereas seven ST23 (63.6%, 7/11) cases were identified as CAIs, indicating the performance of the different infection control strategies. Six ST218 cases (54.5%, 6/11) and four ST23 cases (36.4%, 4/11) were associated with CCI ≥ 3. In addition, two ST218- and six ST23-infected patients had antimicrobial agent exposure within 90 days. Four patients (36.4%, 4/11) with ST218 Kp infection had invasive intubation, and two ST23 (18.1%, 2/11)-infected patients had a drainage tube. One patient experienced metastatic infection in both groups (Table 2).

All eleven ST218 strains in our hospital presented capsule type KL57, and all eleven ST23 isolates possessed KL1. Notably, KL57 and KL1 are closely associated with hypervirulence [1]. A majority of the ST218 isolates (90.9%, 10/11) were confirmed as O2v2 except one with O2v1, and all the ST23 isolates presented O2v2. In terms of the virulence genes, all of the ST218 and ST23 Kp strains harbored *iucA, rmpA, rmpA2, iroB, peg344* and *peg589*, suggesting that they all belong to hypervirulent clones. Notably, all the ST23 strains but none of the ST218 strains presented colibactin-associated genes. Moreover, all the ST23 Kp strains harbored yersiniabactin-associated genes, which were present in seven ST218 Kp isolates (63.6%). For the plasmid types, all ST218 and ST23 Kp isolates harbored IncFIB-type plasmid replicons. None of the ST218 and all ST23 Kp strains harbored the IncHI1B-type plasmid replicon (Additional file 2: Figure S2, Table S2). All eleven ST23 isolates had a virulence score of 5. In contrast, seven and four ST218 strains had virulence scores of 4 and 3, respectively. ST218 and ST23 both belonged to the hypervirulent clone. Importantly, the PEKP3107 carried *bla*_NDM-1_ had a resistance score = 2, while all the other isolates showed a resistance score = 0, suggesting that ST218 tends to acquire carbapenemases (Additional file 2: Table S2).

We selected four Kp isolates, PEKP3107, PEKP2044, 219 and PEKP1095 (ST23), for further phenotype assessments. The mucoviscosity values of PEKP3107, PEKP2044, 219 and PEKP1095 (ST23 as control) were 0.18, 0.11, 0.18 and 0.27, respectively. All four Kp strains possessed strong biofilm-forming capacity and represented sensitivity to serum (Additional file 2: Table S3, Figure S3). Additionally, the growth curve showed no significant difference among the four isolates, suggesting no significant fitness cost of ST218 harboring *bla*_NDM-1_ (Additional file 2: Figure S4). Additionally, the mortality of infected *G. mellonella* larvae indicated no significant difference between the ST218 and ST23 Kp strains, suggesting similar hypervirulence in vivo (Additional file 2: Figure S5).

## Discussion

To the best of our knowledge, this is the first report systematically demonstrating the clinical and genomic characteristics of ST218 Kp, a single-locus variant of ST23, which is commonly identified as hvKp. Notably, we reported the emergence of MDR-ST218-hvKp, which harbored key virulence-associated genes and conferred resistance to carbapenems, β-lactams, quinolones and tetracyclines due to the acquisition of a transferable MDR plasmid located with *bla*_NDM-1_, suggesting that enhanced genomic surveillance is urgently needed.

ST218 was a single-locus variant of ST23, both of which belonged to clonal Group 23 (CG23). This clone frequently presents with the pLVPK-like plasmid [32]. In this study, we found that all of the ST218 strains harbored five key virulence genes, *iroB + iucA + peg344 + rmpA* + *rmpA2* [33]. ST218 is typically susceptible to most antibiotics, and few studies have reported its carbapenem resistance. A previous study showed that ST218 hvKp strains acquired *bla*_OXA-48_ [34]. Another study indicated that the carbapenem-resistant ST218 contributed to the acquisition of *bla*_KPC_ during in vivo evolution [35]. Here, we first reported the acquisition of *bla*_NDM-1_ conferring carbapenem resistance in ST218 hvKp strains. Additionally, ST218 Kp also obtained other resistance genes, *dfrA14*, *qnrS1* and *tet(A),* that formed the MDR-hvKp phenotype. Moreover, our previous study showed that the IS*26* element contributed to the acquisition of resistance genes and the formation of MDR-hvKp [12, 33]. Importantly, the resistance genes *bla*_NDM-1_ and *qnrS1* were flanked by IS26, indicating that enhanced genomic surveillance is essential to prevent recombination and spread. Fortunately, the difference in SNPs among ST218 strains varied more, suggesting sporadic infections within our hospital. Combined with 90 days of antibiotic exposure, the formation of MDR-ST218-hvKp might be associated with antibiotic selective pressure. It is crucial to implement active genomic surveillance to prevent MDR pathogen emergency.

Additionally, we systematically reported the clinical, microbiological and genomic features of the ST218 clone compared with those of the ST23 hypervirulent clone. ST23 is defined as the classical hypervirulent clone mainly attributed to the acquisition of the pLVPK plasmid and is closely associated with pyogenic liver abscess and bloodstream infection [36, 37]. Although most of the ST218 Kp harbored the pLVPK-like plasmid, the main infection type was respiratory infection, suggesting that clinicians should be concerned about the emergence of the ST218 hypervirulent clone. Importantly, ST218 successfully acquired transferable *bla*_NDM-1_ and became MDR-hvKp. Similarly, the convergence of MDR and hypervirulence within ST23 was also reported [33, 38], suggesting that clinicians should be aware of MDR phenotype emergence within hypervirulent clones that hinder antibacterial treatment.

In conclusion, we reported the acquisition of *bla*_NDM-1_ by typical hvKp ST218, presenting both hypervirulence and MDR phenotype, for the first time. The resistance plasmid carrying *bla*_NDM-1_ was transferable. It is of great urgency to enhance genomic surveillance to prevent their rapid propagation and evolution.

### Supplementary Information

**Additional file 1: Table S1.** The differences in SNPs of ST218 *Klebsiella pneumoniae* strains.

**Additional file 2: Table S2.** Clinical and genomic features of ST218 vs ST23 Kp. **Table S3.** Microbiological phenotypes of ST218 vs ST23 Kp. **Figure S1.** Circular sketch map (A) and alignments (B) of plasmid ppekp2044–41. The matched regions between two sequences are displayed by light blue blocks, and the identities are marked (red: antimicrobial resistance genes; green: integrase, recombinase, and transposase genes; purple: transfer associated genes; blue: plasmid replication and conjugation; gray: genes of other functions). **Figure S2.** Genomic distribution of ST218 vs. ST23 *Klebsiella pneumoniae* strains. Red: Virulence genes; Blue: plasmid types; Dark gray: antimicrobial resistance genes. **Figure S3.** Serum killing of PEKP3107, PEKP2044, 219 and PEKP1095. **Figure S4.** Growth curve of PEKP3107, PEKP2044, 219 and PEKP1095. **Figure S5.** Virulence of PEKP3107, PEKP2044, 219 and PEKP1095 assessed by the *Galleria mellonella* infection model.

## Data Availability

All *Klebsiella pneumoniae* genome sequences in this study have been deposited in the NCBI GenBank database under the accession number PRJNA957912. The datasets used and/or analyzed during the current study are available from the corresponding author on reasonable request.

## Abbreviations

hvKp: Hypervirulent *Klebsiella pneumoniae*
cKp: Classical *Klebsiella pneumoniae*
MDR: Multidrug resistant
MDR-hvKp: Multidrug resistant and hypervirulent Kp
CCI: Charlson comorbidity index
SOFA: Sequential organ failure assessment
CAIs: Community-acquired infections
HAIs: Hospital-acquired infections
WGS: Whole genome sequencing
SNPs: Single nucleotide polymorphisms
cgSNPs: Core genome SNPs
