# Supplementary material for: ST218 Klebsiella pneumoniae became a high-risk clone for multidrug resistance and hypervirulence
Source: BMC Microbiol. 2024 Feb 12;24:56. doi: 10.1186/s12866-024-03205-8 (PMC10860259; doi:10.1186/s12866-024-03205-8)

Table S2. Clinical and genomic features of ST218 vs ST23 Kp.

	ST218(n=11)	ST23(n=11)	P-value
**Clinical features**			
Respiratory infection	8(72.7%)	2(18.2%)	0.030
Bloodstream infection	0(0.0%)	8(72.7%)	0.001
Hospital-acquired infection	6(54.5%)	4(36.4%)	0.670
CCI≥3	6(54.5%)	4(36.4%)	0.670
Antimicrobial agent exposure within 90 days	2(18.2%)	6(54.5%)	0.183
Invasive intubation	4(36.4%)	2(18.2%)	0.635
Metastatic infection	1(9.1%)	1(9.1%)	1.000
**Genomic features**			
KL57	11(100.0%)	0(0.0%)	0.000
KL1	0(0.0%)	11(100.0%)	0.000
O2v2	10(90.9%)	11(100.0%)	1.000
Colibactin-associated genes	0(0.0%)	11(100.0%)	0.000
Yersiniabactin-associated genes	7(63.6%)	11(100.0%)	0.090
IncFIB-type plasmid replicon	11(100.0%)	11(100.0%)	1.000
IncHI1B-type plasmid replicon	0(0.0%)	11(100.0%)	0.000
Virulence score=5	0(0.0%)	11(100.0%)	0.000
Resistance score=2	1(9.1%)	0(0.0%)	1.000

Table S3. Microbiological phenotypes of ST218 vs ST23 Kp.

	PEKP3107	PEKP2044	219	PEKP1095
Mucoviscosity	0.18	0.11	0.18	0.27
Biofilm-forming capacity	Strong	Strong	Strong	Strong
Serum killing	Sensitive	Sensitive	Sensitive	Sensitive

Figure S1. Circular sketch map (A) and alignments (B) of plasmid pPEKP2044-41. The matched regions between two sequences are displayed by light blue blocks, and the identities are marked (red: antimicrobial resistance genes; green: integrase, recombinase, and transposase genes; purple: transfer associated genes; blue: plasmid replication and conjugation; gray: genes of other functions).

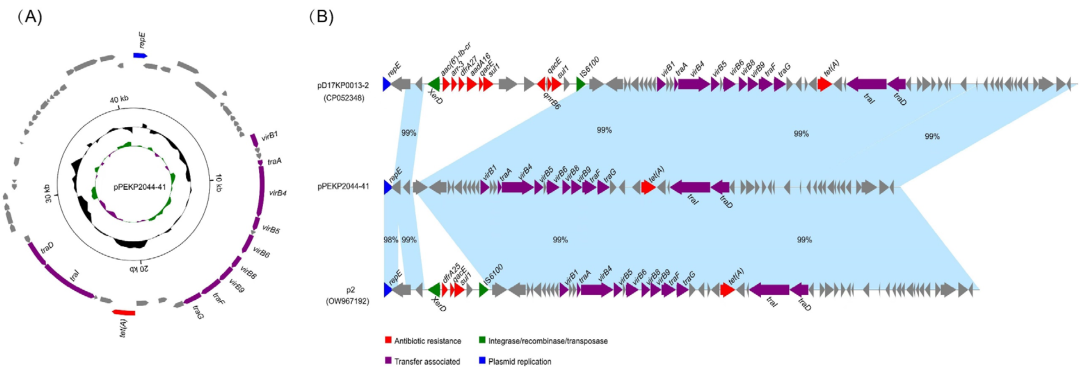

Figure S2. Genomic distribution of ST218 vs. ST23 Klebsiella pneumoniae strains. Red: Virulence genes; Blue: plasmid types; Dark gray: antimicrobial resistance genes.

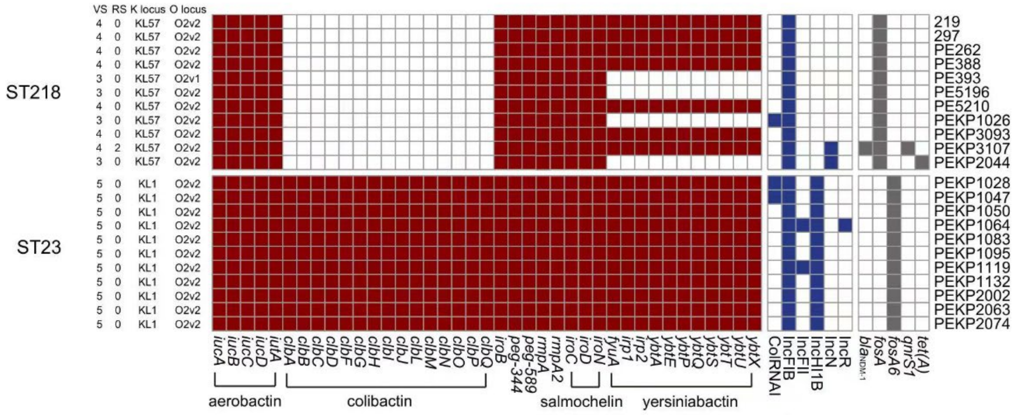

Figure S3. Serum killing of PEKP3107, PEKP2044, 219 and PEKP1095.

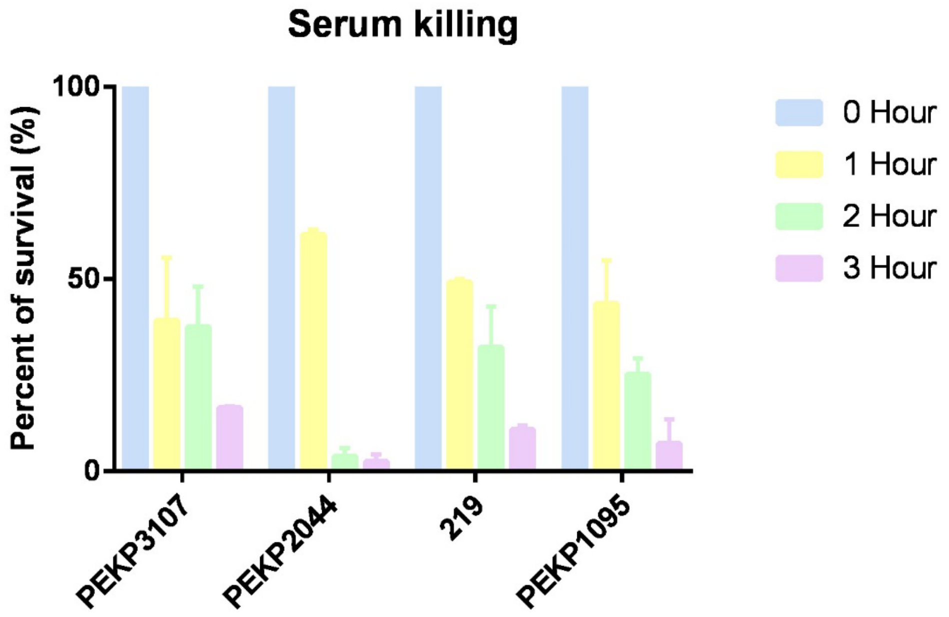

Figure S4. Growth curve of PEKP3107, PEKP2044, 219 and PEKP1095.

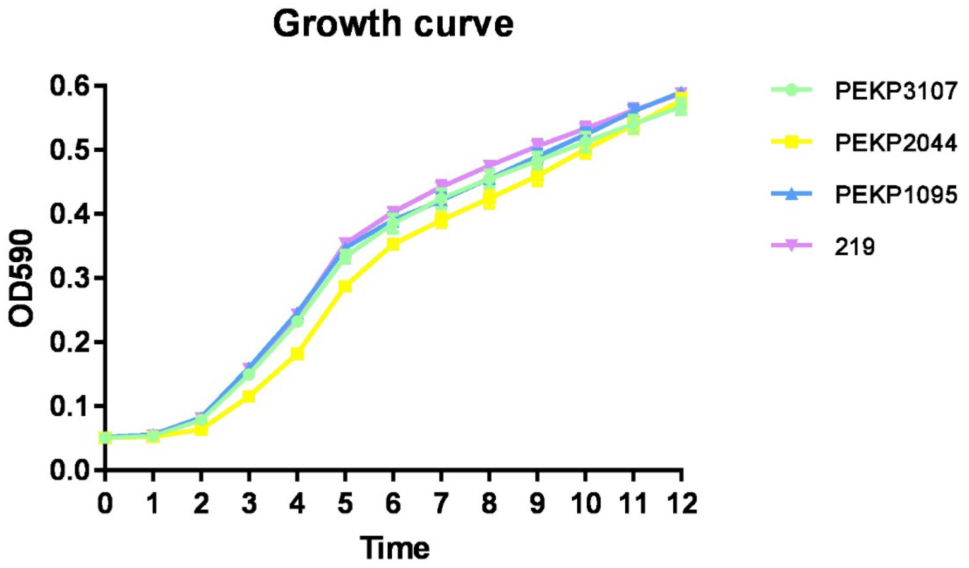

Figure S5. Virulence of PEKP3107, PEKP2044, 219 and PEKP1095 assessed by the *Galleria mellonella* infection model.

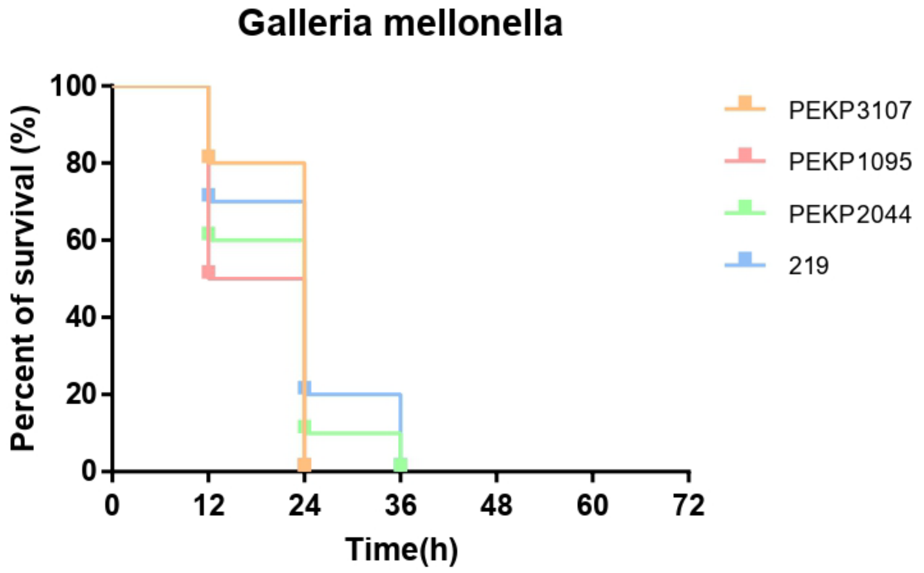

Supplement: Supplementary file 2 — Additional file 2: Table S2. Clinical and genomic features of ST218 vs ST23 Kp. Table S3. Microbiological phenotypes of ST218 vs ST23 Kp. Figure S1. Circular sketch map (A) and alignments (B) of plasmid ppekp2044–41. The matched regions between two sequences are displayed by light blue blocks, and the identities are marked (red: antimicrobial resistance genes; green: integrase, recombinase, and transposase genes; purple: transfer associated genes; blue: plasmid replication and conjugation; gray: genes of other functions). Figure S2. Genomic distribution of ST218 vs. ST23 Klebsiella pneumoniae strains. Red: Virulence genes; Blue: plasmid types; Dark gray: antimicrobial resistance genes. Figure S3. Serum killing of PEKP3107, PEKP2044, 219 and PEKP1095. Figure S4. Growth curve of PEKP3107, PEKP2044, 219 and PEKP1095. Figure S5. Virulence of PEKP3107, PEKP2044, 219 and PEKP1095 assessed by the Galleria mellonella infection model. [file 12866_2024_3205_MOESM2_ESM.pdf]
